# Linking camera‐trap data to taxonomy: Identifying photographs of morphologically similar chipmunks

**DOI:** 10.1002/ece3.7801

**Published:** 2021-06-21

**Authors:** Fiona E. McKibben, Jennifer K. Frey

**Affiliations:** ^1^ Department of Fish, Wildlife and Conservation Ecology New Mexico State University Las Cruces NM USA

**Keywords:** gray‐footed chipmunk, misidentification, *Neotamias canipes*, *Neotamias minimus atristriatus*, Peñasco least chipmunk, remote camera

## Abstract

Remote cameras are a common method for surveying wildlife and recently have been promoted for implementing large‐scale regional biodiversity monitoring programs. The use of camera‐trap data depends on the correct identification of animals captured in the photographs, yet misidentification rates can be high, especially when morphologically similar species co‐occur, and this can lead to faulty inferences and hinder conservation efforts. Correct identification is dependent on diagnosable taxonomic characters, photograph quality, and the experience and training of the observer. However, keys rooted in taxonomy are rarely used for the identification of camera‐trap images and error rates are rarely assessed, even when morphologically similar species are present in the study area. We tested a method for ensuring high identification accuracy using two sympatric and morphologically similar chipmunk (*Neotamias*) species as a case study. We hypothesized that the identification accuracy would improve with use of the identification key and with observer training, resulting in higher levels of observer confidence and higher levels of agreement among observers. We developed an identification key and tested identification accuracy based on photographs of verified museum specimens. Our results supported predictions for each of these hypotheses. In addition, we validated the method in the field by comparing remote‐camera data with live‐trapping data. We recommend use of these methods to evaluate error rates and to exclude ambiguous records in camera‐trap datasets. We urge that ensuring correct and scientifically defensible species identifications is incumbent on researchers and should be incorporated into the camera‐trap workflow.

## Graphical abstract

Misidentification of animals in camera‐trap data can be high even among experts, compromising its utility. We tested a method for ensuring high identification accuracy using two sympatric and morphologically similar chipmunk (*Neotamias*) species as a case study. We recommend use of these methods to evaluate error rates and to remove ambiguous records in camera‐trap datasets, and we urge that ensuring correct and scientifically defensible species identifications is incumbent on researchers and should be incorporated into the camera‐trap workflow.

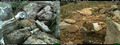

## 1 INTRODUCTION

Camera trapping is becoming a globally widespread technique for surveying and monitoring wildlife populations (Burton et al., 2015; Caravaggi et al., 2017; Wearn & Glover‐Kapfer, 2019). Camera‐traps have advantages over many other survey methods in that they are minimally invasive (Long et al., 2008), are easily deployed, can be left in the field for extended time periods, and can detect rare and elusive species (McShea et al., 2016). Because of these advantages, remote‐camera trapping is a valuable technique for investigating complex questions pertaining to demographics, behavior, and species distributions (Burton et al., 2015; Frey et al., 2017; Gardner et al., 2010). Most recently, camera traps have emerged as an important tool for studying entire communities of mammals (Rich et al., 2016; Tobler et al., 2015) and developing large‐scale biodiversity monitoring networks (McShea et al., 2016; Steenweg et al., 2017).

The use of camera‐trap data depends on the correct identification of animals captured in photographs. However, misidentifications are possible, especially when photograph quality is poor or observers are inexperienced or untrained (Gooliaff & Hodges, 2018; McShea et al., 2016; Meek et al., 2013; Swanson et al., 2016; Thornton et al., 2019). This issue is compounded when sympatric species have similar appearance, and even experts do not always accurately identify species from photographs when morphologically similar species co‐occur (Austen et al., 2016, 2018; Gooliaff & Hodges, 2018; Meek et al., 2013). While studies have investigated error in identifications of camera‐trap photographs, most studies have considered agreement between experts or compared the identification abilities of novices to experts, but did not directly test the ability of observers to identify species through comparison with verified identification (Austen et al., 2016, 2018; Burns et al., 2017; Gooliaff & Hodges, 2018; Thornton et al., 2019).

Many camera‐trap studies target rare species, yet rare species can have both higher false‐positive and false‐negative rates than common species, especially when morphologically similar species co‐occur (Farmer et al., 2012; McKelvey et al., 2008; Swanson et al., 2016). False‐positive errors can lead to overestimations of a species’ distribution or abundance, while false‐negative errors can mean that a subpopulation or habitat type is overlooked (Mackenzie et al., 2002; Royle & Link, 2006). Both types of error may strongly influence conservation outcomes, either by focusing efforts in areas where the species of concern does not occur or by leaving critical subpopulations out of conservation plans. Nonetheless, studies rarely report identification techniques, accuracy rates, or the impact of potential errors on conservation and management plans (Kays et al., 2020; Rich et al., 2016; Tabak  et al., 2018).

Species identifications derive from taxonomy (Walter & Winterton, 2007). At its root, taxonomy depends on a direct comparison of unknown specimens to the holotype or type series, whether through visual examination of museum specimens or consideration of written descriptions (ICZN, 1999). Mammalian taxonomic descriptions rely heavily on morphometric measurements, especially of the skull and dentition, while pelage traits are often of secondary importance (Vaughan et al., 2015). The range of variation within a species is not usually evident in the holotype or type series, and so can be missing from taxonomic descriptions (Farber, 1976; Hull, 1965; Levine, 2001); both nongeographic and geographic variations in pelage traits are especially likely to be overlooked.

The work of taxonomists is communicated to other researchers and to the public in two main ways: keys and field guides. Keys simplify the taxonomic characters into digestible couplets, using the most observable or most diagnostic traits, while disregarding other traits (Hagedorn et al., 2010). Complex Boolean statements are used to account for variation within a species or group, but typically do not reflect the full range of variation. Misidentification error rates are rarely reported with keys, but it is likely that error rates are very high, especially when keys are used by novices (Hagedorn et al., 2010; Walter & Winterton, 2007). Field guides simplify taxonomic information, focusing on visible or in‐the‐field diagnoses (Stevenson et al., 2003). Most field guides include brief species accounts paired with illustrations or photographs and simplified keys, designed for easy use by the public. Mammalian field guides are less available than the ubiquitous bird guides, and many do not focus on regional variations, instead spanning larger areas in order to be more broadly marketable (Stevenson et al., 2003). Because keys and field guides originate from taxonomic descriptions, they are often characterized by the same flaws: (a) They focus on only a few characteristics, and (b) they do not fully account for nongeographic or geographic variation in morphological characters.

When ecologists use photographs as evidence of species presence, the veracity of the identification depends on a number of factors, namely the quality of the photograph, the experience and training of the identifier, and the taxonomic evidence that is used to classify the species. Studies have investigated the influence of the quality and context of photographs and the experience and training of the identifier, but have failed to consider what taxonomic evidence is used by the identifier (Gooliaff & Hodges, 2018, 2019; Meek et al., 2013; Thornton et al., 2019). These issues are exacerbated when morphologically similar species occur within a dataset, necessitating high‐quality photographs, trained observers, and rigorous taxonomic evidence.

**FIGURE 1:**
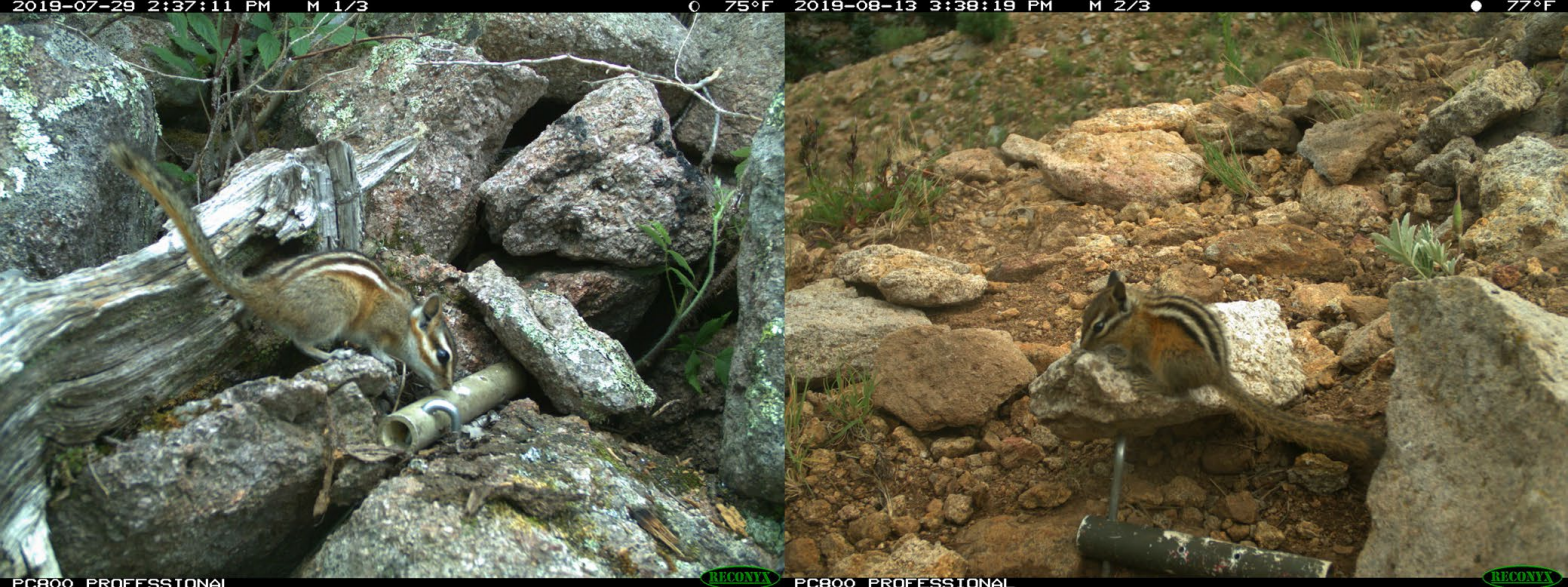
Camera‐trap photographs of *Neotamias canipes* (left) and *Neotamias minimus atristriatus* (right) captured in the Sierra Blanca subrange of the Sacramento Mountains, New Mexico, USA, 2019

Although camera trapping originally was used mainly to study large mammals, the technique is being increasingly used to study other groups of animals that may pose heightened identification problems. For instance, western chipmunks (*Neotamias*) are one of the most diverse groups of small mammals in North America—with many species facing conservation challenges—and yet their morphology is convergent (Patterson, 1981). Researchers have successfully used camera‐traps to study an allopatric population of chipmunk (Perkins‐Taylor & Frey, 2018). However, chipmunk species are often sympatric, posing challenges when using camera traps. For instance, two morphologically similar species of chipmunks occur in the Sacramento Mountains in southern New Mexico, the gray‐footed chipmunk (*N*. *canipes*) and the Peñasco least chipmunk (*N*. *minimus atristriatus*; Figure 1; Best et al., 1992; Verts & Carraway, 2001). The distribution of *N. m*. *atristriatus* has contracted sharply over the last century and it is currently listed as endangered by New Mexico and is a candidate for listing under the Endangered Species Act (USFWS, 2019), while *N*. *canipes* remains common in its range. The ability to monitor the remaining known relict populations of *N. m atristriatus* and survey for new subpopulations using camera‐trapping techniques would have important implications for the management and conservation of this rare subspecies.

Our aim for this study was to develop and test a method for ensuring indisputably high correct identification rates for images obtained via camera trapping. We hypothesized that the accuracy of identifications would improve with a high‐quality identification key and with observer training, when observers identified photographs with higher levels of confidence and when more observers agreed about an identification. To test these hypotheses, we first developed an identification key for distinguishing *N. m*. *atristriatus* and *N*. *canipes* that was based solely on visible pelage traits. We tested the reliability of the key, using verified reference samples, which allowed us to calculate true error rates rather than assessing error through observer agreement. We predicted that error rates would decrease with use of the key versus use of materials in the literature and would decrease with observer training. We predicted that identification accuracy would be correlated with observer confidence and that interobserver agreement would be higher among observers using a key and among observers who were trained in species identification. We assessed the key in a field setting by validating identifications of photographs collected via remote‐camera surveys with results from live‐trapping surveys in the same areas. Through this study, we evaluated a method for identifying morphologically similar species based on photographs that could be adapted for virtually any species.

## 2 METHODS

### 2.1 Development of identification key

We developed and tested an identification key designed to distinguish between *N. m*. *atristriatus* and *N*. *canipes* based solely on pelage traits. To develop the key, we examined museum specimens of each species that had been verified based on analysis of five external, 12 cranial, and 27 pelage measurements (Frey, 2010). There was no significant difference in pelage characters between the sexes (Frey, 2010) and therefore we pooled sexes. We identified 17 pelage traits that appeared to be qualitatively dissimilar between the two species and designed a preliminary identification key that described the differences for each of the 17 traits (Appendix Table A1).

**FIGURE 2:**
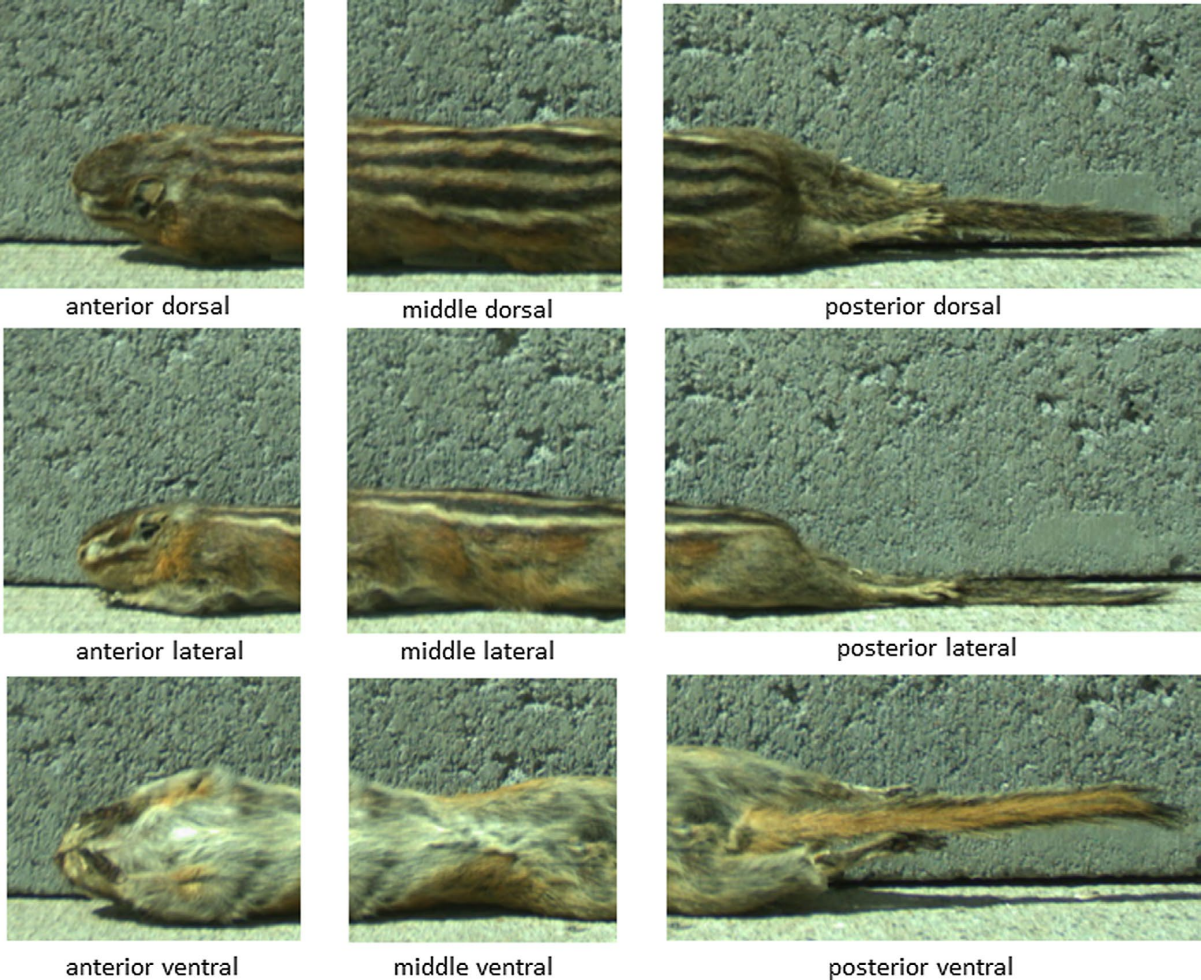
Single *Neotamias minimus atristriatus* specimen divided into nine images or “nonants,” as used for identification key testing and for training materials (see Appendix A)

A laboratory assistant photographed 28 museum specimens of each species using the same type of remote camera (Reconyx PC800 HyperFire, focal distance = 1 m) that would be used in field applications (Appendix Table A2). Specimens were photographed in natural outdoor lighting and positioned in front of a gray background. The camera was set on a surface pointing horizontally, and the museum specimen was positioned 0.5 m away on the same surface. The laboratory assistant photographed each specimen from three angles, rotating the specimen so that either the dorsal, lateral, or ventral side was visible in each photograph. The assistant then subdivided each photograph into three sections (anterior, middle, and posterior), resulting in a total of nine images per specimen, each showing an isolated nonant (i.e., one of nine equally sized sections) of the body (Figure 2). The laboratory assistant randomly ordered all 504 images of nonants as slides in a PowerPoint presentation. The PowerPoint presentation was prepared without direct involvement by the authors to prevent bias.

Each of the authors individually coded every PowerPoint slide for each of the 17 pelage traits (1: best represents trait for *N. m*. *atristriatus*, 2: best represents trait for *N*. *canipes*, and 0: unknown or cannot see feature) and also assigned a species identification to each slide based on our overall impression. In addition, we reported a numeric confidence‐rank from 1 to 4 for each slide, based on our confidence in the attribution of species, from 1: no confidence, 2: not very confident, 3: somewhat confident, and 4: very confident. Because we coded each slide for every visible pelage trait as well as noting an overall impression of the species’ identification, a given pelage trait could be assigned to a different species from the species assigned based on our overall impression. This meant that some traits might be commonly attributed to the wrong species but may not strongly influence the final assessment of species, while others may have a large influence on an overall misidentification. To determine which traits were commonly misidentified and were also contributing to an overall species misidentification, we considered a trait to be “linked to a misidentification” if the trait was attributed to the wrong species and the final assessment of species was also incorrect. We calculated the misidentification rate as the percentage of instances when a trait was linked to a misidentification out of the total instances when the trait was used for an identification.

We examined the misidentification rate for each trait to assess the preliminary identification key and to identify revisions for a final identification key. Using misidentification rates and discrepancies between observers, we improved the trait definitions and developed a final identification key (Appendix Table A3). The final identification key included example comparative photographs of the two species of chipmunk that had been marked to facilitate use of the key.

### 2.2 Evaluating efficacy of the identification key

We tested the efficacy of our final identification key by comparing the accuracy of observers using identification resources from the literature (hereafter, “literature observers”; *N* = 19) to that of observers using our identification key (hereafter, “key observers”; *N* = 15). We provided all observers with Adobe PDF files that included instructions, identification resources, and a test. We provided the literature observers with identification resources that consisted of excerpts from *Mammalian Species* accounts for both species (Best et al., 1992; Verts & Carraway, 2001) and a popular field guide to North American mammals (Reid, 2006). These materials represented the best available identification information attainable without examining specimens. We highlighted sections pertaining to pelage traits to guide observers to the most relevant information for identifications from photographs. We provided the key observers with the identification key. For both groups of observers, the test consisted of 20 slides, each showing three views of a single chipmunk specimen (dorsal, lateral, and ventral). We used three views for testing because in our field applications, cameras fire multiple times providing photographs of an animal from multiple angles—on average, we captured 10.6 photographs of a chipmunk with each visit to a camera and only 7.2% of chipmunk visits to a camera resulted in a single photograph. For each slide, observers recorded a species identification and the numeric confidence‐rank. Observers could only view their own responses during the testing process. The observers were field technicians working on chipmunk field research or undergraduate students in wildlife biology, but they did not have any prior knowledge about chipmunk identification.

We used Welch's unequal variances one‐tailed *t* test to test whether the identification accuracy was higher for key observers than for literature observers. For each group of observers, we calculated the identification accuracy by confidence‐rank and we calculated Pearson's correlation coefficient (*r*) to test for a correlation between confidence‐rank and accuracy. Within groups of observers, we calculated Fleiss’ kappa coefficient (K), which is a measure of interobserver agreement that corrects for how often agreement might happen by chance and ranges from −1 to 1, with 1 indicating perfect agreement and <0 indicating no agreement (Fleiss, 1971).

### 2.3 Investigating the influence of observer training

We tested whether a training program would improve the accuracy of observers who used our identification key. All key observers (*N* = 15) completed the training program. For the training program, observers practiced using the identification key to identify photographs of chipmunk specimens in two separate training sets. After each training set, we provided the trainees with the answer key, so that they could compare their answers to the correct answers and learn from mistakes. The first training set was the original 504 randomized slides showing nonants of specimens of chipmunks, used by the authors for the development of the identification key. The trainees coded each slide for each pelage trait, assigned a species identification based on their overall impression, and reported a numeric confidence‐rank, following the procedure used for the development of the key. The second training set consisted of 168 randomized slides showing a single view (dorsal, lateral, or ventral) of a specimen. For each slide, the trainee assigned a species identification and reported a numeric confidence‐rank. After completing both training sets and reviewing the correct identifications, we considered observers to be fully trained (hereafter “trained key observers”). We tested trained key observers using a post‐training test, which consisted of a set of 56 slides, each showing three views of a single chipmunk specimen (dorsal, lateral, and ventral). For each slide, observers recorded a species identification and the numeric confidence‐rank.

We used a dependent‐samples one‐tailed *t* test to test whether key observers had higher identification accuracy after completing the training program. For the post‐training test, we calculated identification accuracy by confidence‐rank, Pearson's correlation coefficient (*r*) to test for a correlation between confidence‐rank and accuracy, and Fleiss’ kappa coefficient (K). We used a .05 significance level for all tests. We performed statistical analyses and data manipulation using program R 4.0.0 and the irr package (Gamer et al., 2014; R Core Team, 2020).

### 2.4 Field validation of survey results based on image identifications

**FIGURE 3:**
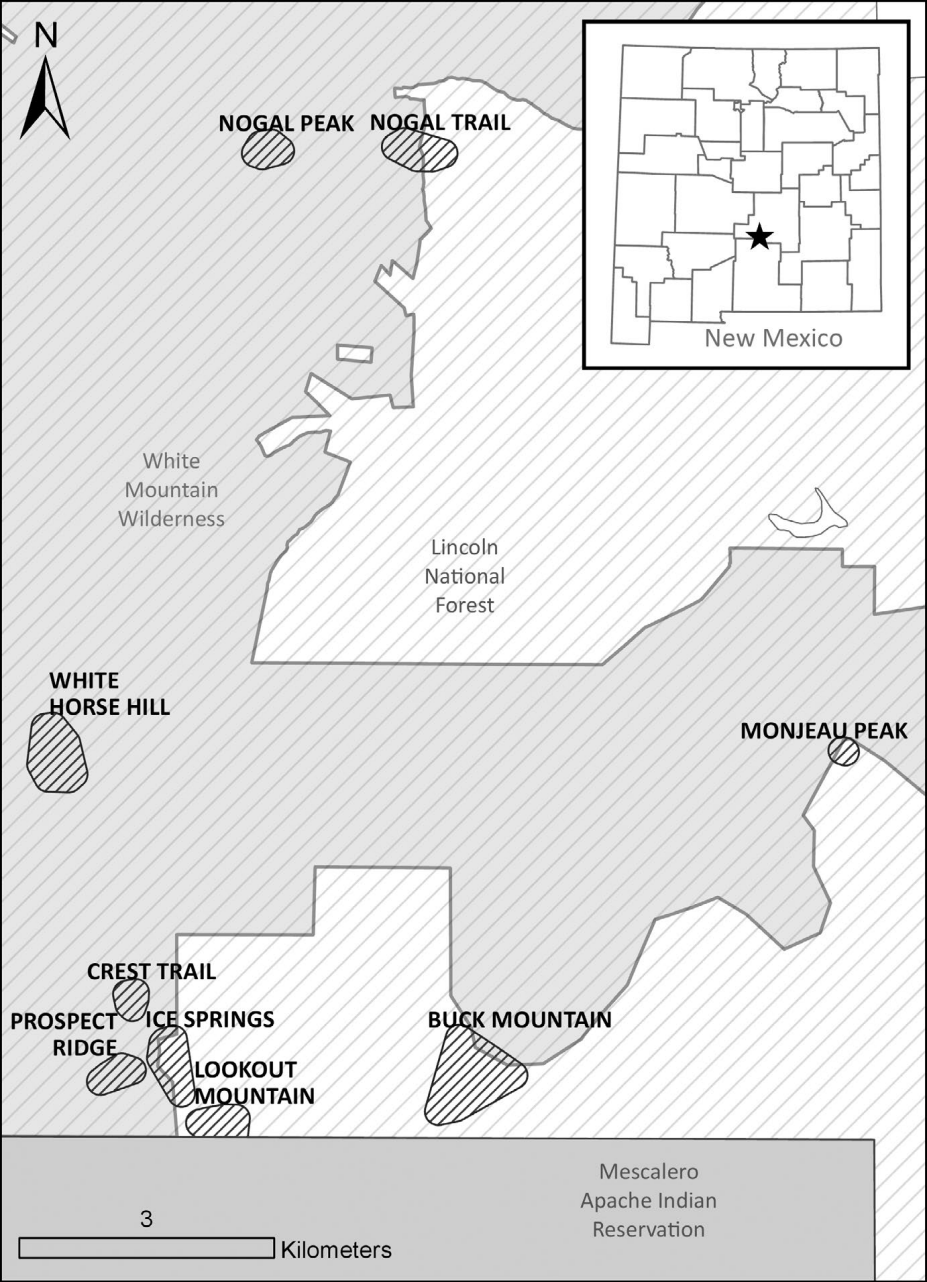
Location of nine field validation study areas in the Sierra Blanca subrange of the Sacramento Mountains, New Mexico, USA, 2018–2019. Chipmunk species detected via Sherman live trapping and camera trapping were compared for each field validation study area (see Table 2). Star in inset map indicates the location of the Sierra Blanca subrange

We conducted surveys for *N. m*. *atristriatus* and *N*. *canipes* using live trapping and camera trapping in nine study areas located in the Sierra Blanca subrange of the Sacramento Mountains, Lincoln National Forest (105°48′56.53″W, 33°23′48.41″N), from 21 June to 17 September 2018 and from 6 June to 7 October 2019 (Figure 3). We validated the camera‐trapping survey results with results of live‐trapping surveys conducted in the same areas (Appendices B and C). The study areas were defined based on a 160 m buffer around a live‐trapping array; the 160 m buffer was based on the diameter of the average home range (ca 2 ha) of *N*. *minimus*, which has the smaller home range of the two species (Bergstrom, 1988; Martinsen, 1968). This ensured that all cameras could potentially fall within the home ranges of chipmunks detected via live‐trapping surveys in the same area.

We identified live‐captured chipmunks using a suite of diagnostic morphological characters, including morphometric measurements and pelage traits (Frey, 2010). Trained observers identified images of chipmunks from the camera traps. We considered photographs of chipmunks as confirmed species identifications if all observers agreed on the species identification and rated the identifications very confident.

## 3 RESULTS

### 3.1 Development of identification key

Using the preliminary identification key, the authors correctly identified 90.7% of the photographs of nonants of specimens (Appendix D). Ventral tail was frequently linked to misidentifications and so it was eliminated in the final identification key. We used differences in coding between the authors to revise the definitions of belly and underside of back leg in the final identification key (Appendix Table A3). Photographs of dorsal and lateral views had higher accuracy rates (91.6% and 92.0%, respectively) than photographs of ventral views (88.3%), so we designed a mounting apparatus for our camera traps to capture these angles in the field (Appendix C).

### 3.2 Evaluating efficacy of the identification key

Observers using identification resources from the literature had low accuracy rates (78.2%) and were significantly (*t* = −4.4, *df* = 27.0, *p* < .001) less accurate than key observers (accuracy = 93.0%). Identification accuracy increased with confidence‐rank for observers using the identification key, but there was no clear relationship between accuracy and confidence for literature observers (Table 1). For key observers, accuracy was positively correlated with confidence‐rank (*r* = .91), and when they reported very high confidence (confidence‐rank 4), accuracy was 100%. Fleiss’ kappa coefficient for interobserver agreement was higher for key observers than for literature observers: Literature observers had low agreement (K = 0.47), and key observers had moderate agreement (K = 0.75).

**TABLE 1:** Accuracy of identification of *Neotamias minimus atristriatus* and *Neotamias canipes* from photographs of verified museum specimens at different observer reported confidence‐ranks for literature observers and key observers before and after training

	Observer confidence	Number of identifications	Accuracy (% correct)	Fleiss’ kappa coefficient (K)
Literature observers	No confidence	8	88.9	
	Not very confident	101	68.8	
	Somewhat confident	150	86.1	
	Very confident	81	91.2	
	All confidence‐ranks	340	78.2	0.47
Key observers, before training	No confidence	19	63.3	
	Not very confident	67	89.7	
	Somewhat confident	119	96.1	
	Very confident	95	100.0	
	All confidence‐ranks	300	93.0	0.75
Key observers, after training	No confidence	11	92.0	
	Not very confident	61	96.3	
	Somewhat confident	221	96.9	
	Very confident	491	100.0	
	All confidence‐ranks	784	98.8	0.95

### 3.3 Investigating the influence of observer training

Although key observer accuracy was high before training (93.0%), accuracy increased significantly (*t* = −4.0, *df* = 14, *p* < .001) through the training program to 98.8%. The strength of the correlation between accuracy and confidence‐rank increased with training, from *r* = .91 before training to *r* = .96 after training. When trained key observers reported somewhat or very high confidence (confidence‐rank 3 and 4), accuracy was 99.2%; accuracy was 100% when they had very high confidence (Table 1). Fleiss’ kappa coefficient increased with training, from moderate agreement (K = 0.75) before training to very high agreement (K = 0.95) after training.

### 3.4 Field validation of survey results based on image identifications

The field validation included 11,103 live‐trapping days and 806 camera‐trapping days across the two years. We captured 15,847 photographs of chipmunks on camera traps, and 7,300 of those photographs met the criteria as confirmed species identifications. Of the discarded photographs, 99.3% had at least one observer report a lower confidence‐rank (1, 2 or 3) and 13.0% were identified as both species. At least one observer reported a confidence‐rank of 1 (no confidence) on 5.6% of the discarded photographs, a confidence‐rank of 2 (not very confident) on 27.6% of the discarded photographs, and a confidence‐rank of 3 (somewhat confident) on 89.3% of the discarded photographs. At eight of the nine field validation study areas, we detected the same species using both methods (Table 2). At the Crest Trail study area, we captured a single *N*. *canipes* via live trapping, while no chipmunks were detected on camera.

**TABLE 2:** Results from surveys at nine field validation sites, comparing chipmunk species detected via live trapping and via camera trapping in the Sierra Blanca subrange of the Sacramento Mountains, New Mexico, USA, 2018–2019. A check mark indicates that the species was detected at least once using a given detection method, and ‐‐ indicates that the species was not detected

	Sherman live trap detections			Camera‐trap detections		
Sites	Trap days	*Neotamias minimus atristriatus*	*Neotamias canipes*	Camera days	*Neotamias* *minimus atristriatus*	*Neotamias canipes*
Ice Springs	2,076	**✓**	**✓**	171	**✓**	**✓**
Prospect Ridge	255	**‐‐**	**‐‐**	76	**‐‐**	**‐‐**
Crest Trail	340	**‐‐**	**✓**	86	**‐‐**	**‐‐**
Lookout Mountain	3,142	**✓**	**✓**	71	**✓**	**✓**
Buck Mountain	750	**‐‐**	**✓**	167	**‐‐**	**✓**
Monjeau Peak	500	**‐‐**	**✓**	68	**‐‐**	**✓**
White Horse Hill	680	**‐‐**	**‐‐**	58	**‐‐**	**‐‐**
Nogal Peak	1,440	**✓**	**✓**	87	**✓**	**✓**
Nogal Trailhead	1,920	**‐‐**	**✓**	22	**‐‐**	**✓**

## 4 DISCUSSION

### 4.1 Key findings

Through a carefully controlled process, we demonstrated highly reliable identifications of two cryptic species of chipmunk based on images obtained via remote cameras. Identification rates improved from low accuracy (78.2%) by observers using literature references to nearly perfect accuracy (98.8% overall or 100% when reporting very high confidence) by trained observers using a specifically developed identification key. Many past studies of misidentification using camera traps measured rates of disagreement among experts (Austen et al., 2018; Gooliaff & Hodges, 2018) or between novices and experts (Burns et al., 2017), while our evaluation compared identifications to verified reference samples. The comparison of identification with known samples enabled us to report true error rates. Because we trained our observers to self‐evaluate their identification abilities, when a photograph was low quality or captured poor ambient light conditions, the observers assigned a low confidence‐rank. Observer confidence‐rank and observer agreement were inversely related to error rate, so we had an error‐linked basis for excluding ambiguous records from the database. The entire process guaranteed that our final database had indisputably low error rates.

### 4.2 Conservation implications of misidentification in camera trapping

The use of camera traps is widespread (Wearn & Glover‐Kapfer, 2019), but a more rigorous examination of the foundation of species identifications is needed. Even expert identifications can have high error rates (Gibbon et al., 2015; Meek et al., 2013), yet many studies do not provide information on how identifications were made (Kays et al., 2020; Rich et al., 2016; Steenweg et al., 2016). Most studies consider expert identification to be the gold standard (Swanson et al., 2016), yet Meek et al. (2013) found that experts had very low accuracy (44.5%) when identifying small‐ and medium‐sized mammals from camera‐trap photographs when morphologically similar species co‐occurred. Species experts also disagreed on identifications of Canada lynx (*Lynx canadensis*) and bobcats (*Lynx rufus*; Gooliaff & Hodges, 2018), bumblebees (*Bombus* sp.; Austen et al., 2016), and newts (Austen et al., 2018). While some studies provided training and reference materials to inexpert observers, the training materials were not assessed, the experts were not trained, and the expert identifications were unquestioned (e.g., Burns et al., 2017; Thornton et al., 2019). Many experts in the fields of ecology and wildlife management are experts on the ecology and management of their study species, rather than experts in the species’ taxonomy (Thornton et al., 2019). Strikingly, Farmer et al. (2012) found that experts are more confident in their species identifications than nonexperts, but observers of all skill levels are equally overconfident or equally as likely to wrongly believe that their identifications are error‐free.

Uncertainty in camera‐trap datasets is often ignored. Even species with otherwise obvious distinguishing characteristics can be misidentified by experts if photograph quality is poor or odd angles are captured, yet researchers rarely report how mediocre a photograph must be or the confidence of the identification necessary to merit removal from the dataset (King et al., 2020). Meek et al. (2012) explicitly managed the uncertainty in their dataset by classifying detections as “probable” or “definite,” but most studies completely ignore ambiguity in identifications (e.g., Tobler et al., 2008). Often researchers deal with uncertainty by soliciting identifications from multiple observers and defaulting to the opinion of the majority (e.g., Gooliaff & Hodges, 2018; McShea et al., 2016; Swanson et al., 2016). We wonder why this system is so widely used, when it is evident that if trained or expert observers do not agree on an identification, then the record is questionable. Studies seldom report error rates, which makes it impossible to impartially judge the reliability of results or inferences, and field validations that might alleviate ambiguity are rarely undertaken (Ladle et al., 2018; Mills et al., 2019; Steenweg et al., 2016). A review of the camera‐trap literature reveals that in studies of multispecies assemblages in which misidentifications are possible, researchers rarely report identification error rates, observer training procedures, or the methods used to remove ambiguous photographs from the database (Kays et al., 2020; Rich et al., 2016; Rowcliffe et al., 2014; Tabak et al., 2018; Tobler et al., 2008). Our methods directly address these issues by explicitly linking error to confidence and observer agreement, providing evidence‐based criteria for minimizing uncertainty in databases.

Misidentification is an especial concern for rare and elusive species, understudied species, and species of conservation concern, especially when these species co‐occur with morphologically similar species. Swanson et al. (2016) found that species that were rare in their dataset had both higher false‐positive and false‐negative rates than common species, likely because observers were eager to report rare species and because common species provided more opportunities for learning (although observers classified some species with high accuracy regardless of rarity, probably due to distinctive traits). Similarly, in a brief analysis wherein we created unbalanced sets of slides of each chipmunk species, we confirmed that rarity was associated with lower identification accuracy (Appendix F). Species might be rare in a dataset because they are rare on the landscape, are rare at surveyed sites, or are especially elusive to detection; regardless, false positives can have overblown impacts on parameters of interest for rare species (Swanson et al., 2016). Understudied and imperiled species are often rare, difficult to detect (Linkie et al., 2013; Thomas et al., 2020), and vulnerable to mismanagement, and so ensuring high identification accuracy for these species is of especial importance.

The impacts of misidentifications in camera‐trap studies remain mainly unaddressed. Misidentifications can lead to faulty inferences, such as errors in estimates of species distributions, community structure and dynamics, or extinction/colonization rates. Like any questionable occurrence records, misidentified camera‐trap data can hinder appropriate conservation actions (Aubry et al., 2007), lead to a misallocation of resources, putatively resurrect extinct species (McKelvey et al., 2008), and even lead to supposed discoveries of entirely new species (Meijaard et al., 2006). Management based on faulty inference can be expensive and wasteful (McKelvey et al., 2008) and can be open to legal disputes. The US federal government spent nearly $6,000,000 conserving habitat for the ivory‐billed woodpecker (*Campephilus principalis*), which was considered to be extant based on a four‐second blurry video (Jackson, 2006; USFWS, 2006), while federal protection for the fisher (*Pekania pennanti*) in western North America was delayed because questionable records indicated that the species was wide‐ranging (McKelvey et al., 2008). Because camera‐trap photograph identifications are rarely confirmed, it is unknown how much money and effort has been similarly wasted and misallocated due to these misidentifications. Given the upsurge in remote‐camera surveys worldwide (Wearn & Glover‐Kapfer, 2019), the deployment of remote cameras in biodiversity monitoring networks that require identifications of many species (Kays et al., 2020; Steenweg et al., 2017), and the increased use of camera traps for taxonomic groups that commonly co‐occur with morphologically similar species (De Bondi et al., 2010; McDonald et al., 2015; Perkins‐Taylor & Frey, 2020), both the risk of misidentification and the impacts on global conservation will increase if unaddressed.

### 4.3 Recommendations for camera‐trap studies involving morphologically similar species

Our stringent methods allowed us to assure indisputably high correct identification rates, but this also required significant time and labor. We estimate that the process to develop a key, train the observers, and test the efficacy of the key required >195 hr, exclusive of the time required to verify the identity of the reference specimens (Appendix G). Additional labor also was incurred by the need to have three trained observers review and code all photographs from the field. Regardless, we considered these investment as necessary because (a) the species were extremely difficult to differentiate, (b) there was little existing information on the nature and variation of external diagnostic characters, (c) the target species was rare and thus more susceptible to misidentifications, (d) the target species was a species of conservation concern, with high potential impacts of misidentification, (e) we planned to use our method to investigate occupancy of the target species, and parameters in occupancy models are sensitive to misidentifications, and (f) policy makers and managers will need to have confidence in future research findings using these methods to investigate the target species.

We recommend that other studies follow our methods when there are similar concerns. However, given the significant labor involved in the process, we acknowledge that not all of our methods are necessary for all camera‐trap studies and that this will depend on the study goals and species involved. As a piece of the study design phase, researchers need to consider (a) are misidentifications likely? (b) are there well‐developed data available on diagnostic traits and their variability? (c) will misidentifications affect parameter estimates and management or conservation outcomes? Researchers can use these questions to determine an acceptable error rate for their study, to estimate the labor costs, and to determine whether our stringent methods are necessary or whether an abbreviated version of our methods would be sufficient to meet project goals.

We recommend a sliding scale of identification methods, grading from the most stringent methods, necessary in studies such as ours, to the simplest methods, which represent the bare minimum to be used in all camera‐trap studies (Table 3). In stringent cases, we recommend that researchers perform the entire key creation and verification process using verified reference samples, provide extensive observer training, use multiple observers to identify species, and record confidence‐ranks with identifications. These studies should report the key, details of the training process, error rates by confidence‐rank from the training process, and what threshold of confidence and agreement was used to omit photographs from the final database. In studies of morphologically similar species that are well‐studied and easier to differentiate, we recommend that researchers follow an abbreviated version of our methods (Table 3). This applies to species such as lynx and bobcat, because (a) misidentifications are likely (Gooliaff & Hodges, 2018), (b) there is a consensus on at least some diagnostic traits, and (c) one of the species is of conservation concern (USFWS, 2000). In such situations, extensive key development may not be necessary because diagnostic traits are well established and the training process can be abbreviated; however, researchers should still train and test observers using verified reference samples (either with verified museum specimens or with verified photographs), report error rates, and use confidence and observer agreement to omit ambiguous photographs. Lastly, at a bare minimum, we recommend that researchers follow the simplest version of our identification methods (Table 3). These methods apply when study species are easily differentiated (e.g., elephant versus giraffe) and the impacts of a false positive on conservation and management outcomes are deemed to be low. In such situations, observers should be supplied with a list of target species and basic identification information (e.g., photograph examples), identifications should include a simple confidence‐rank (e.g., “sure” versus “unsure”), and researchers should report the methods used to omit ambiguous photographs (McShea et al., 2016). By following these recommendations, researchers can ensure that identifications in their final database are scientifically defensible.

**TABLE 3:** Recommended steps for the identification process in camera‐trap studies. Check marks indicate that we recommend a step should be followed under that method. We recommend the simple method when study species are easily differentiated and the impacts of a false positive on conservation and management outcomes are deemed to be low. We recommend abbreviated methods when misidentifications are likely, there is a consensus on diagnostic traits, and the target species is of conservation concern. We recommend stringent methods when species are difficult to differentiate, there is little information on diagnostic traits, and the target species is of conservation concern

Overview	Steps		Method	
		Simple	Abbreviated	Stringent
Create a key based on external characteristics	1) Examine verified specimens or verified photographs to identify potential differentiating pelage traits or other external characteristics	**‐‐**	**✓** ^a^	**✓**
	2) Create a key based on external characteristics	**✓**	**✓**	**✓**
	3) Test key to ensure it is possible to differentiate species with a reasonable level of accuracy	**‐‐**	**‐‐**	**✓**
	4) Revise key based on test results in order to improve its efficacy	**‐‐**	**‐‐**	**✓**
Train observers on use of key and use of confidence‐ranks	1) Observers practice identification and confidence ranking using randomized photographs of all possible views (e.g., nonants or quadrants) followed by review of correct identifications	**‐‐**	**✓** ^b^	**✓**
	2) Observers practice identification and confidence ranking using randomized photographs of thirds (dorsal, lateral, ventral) followed by review of correct identifications	**‐‐**	**‐‐**	**✓**
	3) Test observers on identifications with confidence rankings using full body views (or relevant view to be used in field)	**‐‐**	**✓**	**✓**
	4) Identify best camera angle for differentiating the target species	**‐‐**	**✓**	**✓**
	5) Calculate error rates overall, by confidence‐rank, and by agreement level	**‐‐**	**✓**	**✓**
	6) Determine acceptable error rate for confirmed identifications	‐‐	**✓**	**✓**
Implement	1) Collect camera‐trap data (using best camera angle, as identified during training)	**✓**	**✓**	**✓**
	2) Observers identify species in photographs with confidence‐ranks	**✓**	**✓**	**✓**
	3) Omit photographs based on confidence‐rank and agreement level (relate to error rates during training)	**✓**	**✓**	**✓**
	4) Report key	**✓**	**✓**	**✓**
	5) Report details of training process	**‐‐**	**✓**	**✓**
	6) Report relevant error rates	**‐‐**	**✓**	**✓**
	7) Report threshold of confidence‐rank and agreement level used to omit ambiguous photographs	**✓**	**✓**	**✓**

^a^ Review literature to identify potential differentiating characteristics.

^b^ Observers practice on different views, including all possible angles, followed by review of correct identifications.

Undergraduate‐level wildlife and biology courses are increasingly using camera‐trap networks as a teaching tool (Karlin & De La Paz, 2015). Our method could integrate with these courses, with students developing and testing keys, and eventually providing high‐quality identifications based on known error rates. Undergraduate students are commonly used as observers in camera‐trap studies, and integrating these methods within ecology and biology departments would develop cohorts of well‐trained and thoughtful photograph identifiers.

False‐positive models have recently been touted as a solution to uncertain detections, as these models may have lower bias, greater model support, and sometimes result in considerably different parameter estimates (Clare et al., 2020; Miller et al., 2011). However, most of these models couple confirmed (i.e., error‐free) data with ambiguous data, and so use of these techniques does not absolve researchers from the need to make correct identifications. Confirmed detections can be obtained at a subset of sites through independent methods such as live trapping or hair snaring for DNA. In other cases, confirmed detections might be obtained by the verification of a subset of identifications in a camera‐trap dataset (Clare et al., 2020). This requires the ability to make error‐free verifications of identifications. Our methods can facilitate this process. If researchers can identify a threshold of confidence‐rank and observer agreement at which identifications are highly accurate, they can use this to divide the data into “confirmed” and “ambiguous” detections, to be analyzed in a false‐positive model. This eliminates the need for “experts” (who are usually principal investigators) to spend valuable time reviewing identifications made by volunteers and technicians.

Citizen‐science data processing and machine‐learning models have recently been used to streamline and standardize species identifications of large datasets of images (Swanson et al., 2016; Tabak et al., 2018), but these methods do not preclude the need to assess accuracy and eliminate bad records. Recently developed methods provide a framework for training citizen‐scientist volunteers, managing and aggregating volunteer identifications, and verifying those data through expert opinion (McShea et al., 2016). Instead of depending on expert verifications and agreement algorithms (Swanson et al., 2016), these platforms could integrate observer training on species taxonomy, self‐reported confidence‐ranks, and frequent observer testing. This would provide a running estimate of observer accuracy by confidence‐rank and thus facilitate the screening of data for high accuracy records. Machine‐learning methods might also be a valuable tool for identifying morphologically similar species, but model training depends on identifications made by researchers and the models are prone to low accuracy for rare species (Willi et al., 2018). Consequently, we recommend that researchers apply the methods outlined in our study to validate training sets using verified reference samples and evaluate error rates, observer confidence, and agreement. In some situations, machine‐learning methods could be used to screen through multispecies assemblages for species that are difficult to differentiate, identifying the species that require more stringent identification methods.

Regardless of what methods are used to assess and reduce error, all camera‐trap studies should consider and describe the potential impacts of misidentifications on inferences and on conservation and management plans. False positives and false negatives will impact inferences differently, so researchers should consider study goals when choosing rules for inclusion of photographs in the database. For example, researchers interested in species occupancy (Mackenzie et al., 2002) might require a higher level of confidence in identification. While omitting photographs from an occupancy database feels wasteful, researchers should remember that a missed occurrence record due to poor photograph quality can be accounted for by common methods for dealing with imperfect detection (Mackenzie et al., 2002; Royle et al., 2005), while a false‐positive occurrence record will likely lead to faulty inferences (Aubry et al., 2017; McKelvey et al., 2008). Conversely, researchers interested in identifying future survey sites for documenting new populations of a rare species might include lower confidence records. Our method facilitates these processes by assigning confidence‐ranks to identifications. Whatever the goals of the study, it is imperative that researchers consider the potential impacts of misidentifications on all inferences and conservation actions.

## CONFLICT OF INTEREST

We have no conflicts of interest to declare.

## AUTHOR CONTRIBUTIONS

**Fiona E. McKibben:** Data curation (lead); Formal analysis (lead); Investigation (lead); Methodology (equal); Visualization (lead); Writing‐original draft (lead); Writing‐review & editing (equal). **Jennifer K. Frey:** Conceptualization (lead); Funding acquisition (lead); Methodology (equal); Supervision (lead); Writing‐review & editing (equal).

## ETHICAL APPROVAL

The New Mexico State University Office of Research Integrity and Compliance found that the research was exempt from Institutional Review Board review (number 20485). Field methods were approved by the New Mexico State University Institutional Animal Care and Use Committee (number 2018‐005).

## Figures and Tables

**FIGURE C1:**
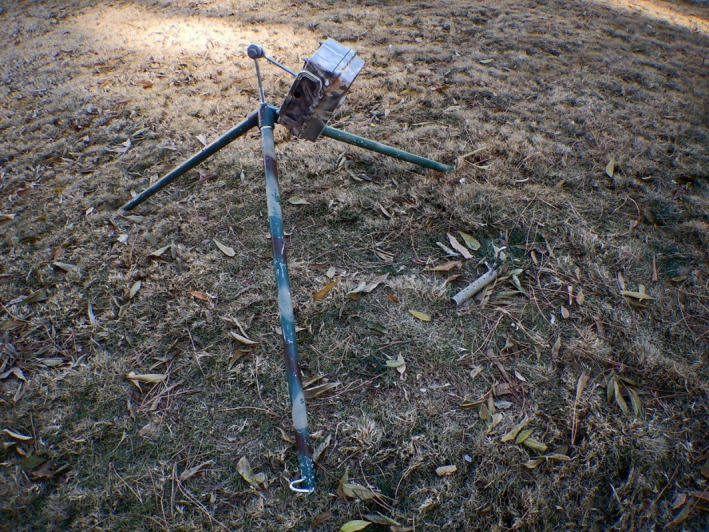
Camera stand and bait tube used to survey Peñasco least chipmunks (*Neotamias minimus atristriatus*) and gray‐footed chipmunks (*Neotamias canipes*) in the Sierra Blanca subrange of the Sacramento Mountains, New Mexico, USA, 2018–2019

**FIGURE C2:**
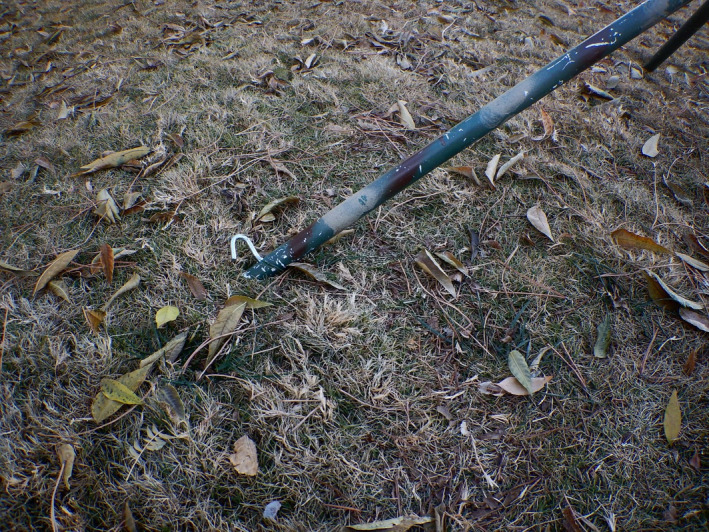
Leg of tripod of camera stand with a tent stake pegged into the ground, as used to survey Peñasco least chipmunks (*Neotamias minimus atristriatus*) and gray‐footed chipmunks (*Neotamias canipes*) in the Sierra Blanca subrange of the Sacramento Mountains, New Mexico, USA, 2018–2019

**FIGURE C3:**
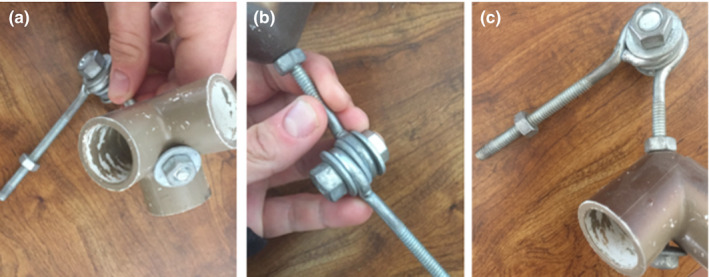
Close up of adjustable camera attachment used to survey Peñasco least chipmunks (*Neotamias minimus atristriatus*) and gray‐footed chipmunks (*Neotamias canipes*) in the Sierra Blanca subrange of the Sacramento Mountains, New Mexico, USA, 2018–2019. (a) Eyebolt attachment to the top of the tripod. (b) Joint between two eyebolts. (c) Free end of eyebolt where camera attaches

**FIGURE C4:**
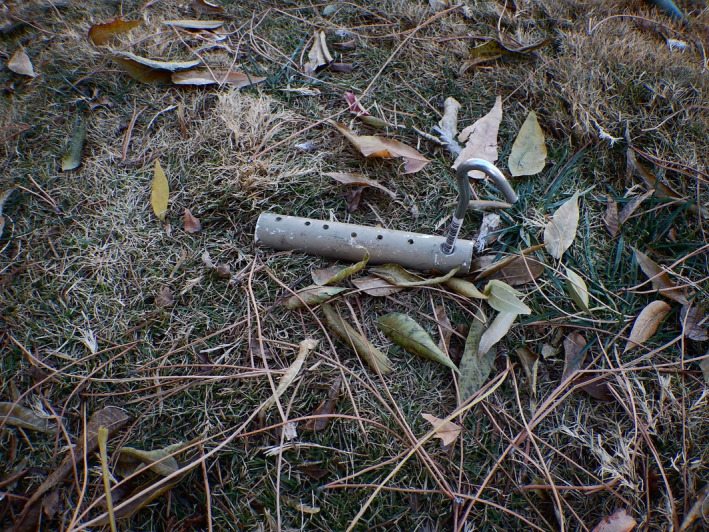
Bait tube used to survey Peñasco least chipmunks (*Neotamias minimus atristriatus*) and gray‐footed chipmunks (*Neotamias canipes*) in the Sierra Blanca subrange of the Sacramento Mountains, New Mexico, USA, 2018–2019

## Data Availability

Data are uploaded to Dryad Digital Repository https://doi.org/10.5061/dryad.kkwh70s4n

## APPENDIX A

### Identification key development

**TABLE A1:** Preliminary identification key based on 17 qualitative pelage traits considered to be potentially useful for distinguishing between *N. m*. *atristriatus* and *N*. *canipes* from photographs. This key was revised and updated following a testing phase (see Table A3 for final identification key)

Pelage trait	*Neotamias minimus atristriatus*	*Neotamias canipes*
Post auricular patches: small patches of lighter fur directly posterior to ears	Small and darker	Larger, prominent and white
Lower face: lighter patch below lowest dark stripe	Dingy or yellowish	Whitish or clean pale gray
Lower light face stripe: light stripe below eye that goes to ear	Grayish or dingy	White
Upper light face stripe: light stripe/patch above eye	Less white, less prominent	White
Crown: top of head	Yellowish, orange, darker	Less orange, lighter
Shoulder	Yellowish, orange, darker, more intense	Grayer, lighter, less intense
Dark outer stripes: there are five dark dorsal stripes—this refers to the pair of outermost stripes, and these stripes may be indistinct	Blacker; narrower and more distinct (looks like it was drawn on with a marker)	Browner; wider and less distinct (looks like it was painted on with a brush)
White outer stripes: there are four light stripes—this refers to the pair of outermost light stripes	Dingy mixed with brown hairs	White
Dark median stripes: the pair of dark stripes immediately lateral to the middle dark stripe	Darker, thin, blackish (looks like it was drawn on with a marker)	Thick, brownish (looks like it was painted on with a brush)
Dark stripes on rump: this character describes whether the pair of dark median stripes changes color over the rump	The pair of dark median stripes remains dark and distinct all the way down over the rump to near the base of the tail	The pair of dark median stripes changes color posteriorly, becoming a lighter brown and may become so indistinct as to disappear
Hip	Yellower/more orange	Gray
Dorsal hindfoot	Pale yellowish orange	Yellowish gray
Dorsal tail	Hairs mixed black and orange	Hairs mixed black and white
Ventral tail	Orange down the center, black edges, orange tipped hairs	Orange down the center, black edges, white tipped hairs
Belly	Light beige, yellowish or orange; darker	Creamy or white; lighter
Underside of back leg	Orange	White/gray
Underside of front leg	Orange	White/gray

**TABLE A2:** List of museum specimens of *Neotamias minimus atristriatus* and *Neotamias canipes* used to create testing and training materials for the development of an identification key for use with camera‐trap photographs. Specimens were borrowed from the New Mexico State University Wildlife Museum (NMSU), the Museum of Comparative Zoology at Harvard (MCZ Harvard), and the Academy of Natural Sciences of Philadelphia (ANSP)

Catalog number	Species
NMSU 2415	*Neotamias canipes*
NMSU 2492	*Neotamias canipes*
NMSU 2479	*Neotamias canipes*
NMSU 2417	*Neotamias canipes*
NMSU 2410	*Neotamias canipes*
MCZ Harvard 24628	*Neotamias canipes*
NMSU FT 875	*Neotamias canipes*
NMSU FT 380	*Neotamias canipes*
MCZ Harvard 24624	*Neotamias canipes*
NMSU FT 874	*Neotamias canipes*
NMSU 2480	*Neotamias canipes*
NMSU 2413	*Neotamias canipes*
NMSU 2411	*Neotamias canipes*
NMSU FT 377	*Neotamias canipes*
NMSU FT 373	*Neotamias canipes*
NMSU FT 378	*Neotamias canipes*
NMSU 2414	*Neotamias canipes*
NMSU 2416	*Neotamias canipes*
MCZ Harvard 24623	*Neotamias canipes*
NMSU FT 379	*Neotamias canipes*
NMSU 2412	*Neotamias canipes*
NMSU 2409	*Neotamias canipes*
NMSU 2418	*Neotamias canipes*
NMSU FT 376	*Neotamias canipes*
NMSU 2491	*Neotamias canipes*
NMSU FT 375	*Neotamias canipes*
NMSU 2478	*Neotamias canipes*
NMSU 2408	*Neotamias canipes*
ANSP 15573	*Neotamias minimus atristriatus*
ANSP 14649	*Neotamias minimus atristriatus*
ANSP 14648	*Neotamias minimus atristriatus*
ANSP 14644	*Neotamias minimus atristriatus*
ANSP 15578	*Neotamias minimus atristriatus*
ANSP 14634	*Neotamias minimus atristriatus*
ANSP 14652	*Neotamias minimus atristriatus*
ANSP 14636	*Neotamias minimus atristriatus*
ANSP 15585	*Neotamias minimus atristriatus*
ANSP 15568	*Neotamias minimus atristriatus*
ANSP 14637	*Neotamias minimus atristriatus*
ANSP 15569	*Neotamias minimus atristriatus*
ANSP 14645	*Neotamias minimus atristriatus*
ANSP 14633	*Neotamias minimus atristriatus*
ANSP 14640	*Neotamias minimus atristriatus*
ANSP 15584	*Neotamias minimus atristriatus*
ANSP 14646	*Neotamias minimus atristriatus*
ANSP 15577	*Neotamias minimus atristriatus*
ANSP 14639	*Neotamias minimus atristriatus*
ANSP 15586	*Neotamias minimus atristriatus*
ANSP 14635	*Neotamias minimus atristriatus*
ANSP 15589	*Neotamias minimus atristriatus*
MCZ Harvard 24613	*Neotamias minimus atristriatus*
ANSP 14642	*Neotamias minimus atristriatus*
ANSP 14641	*Neotamias minimus atristriatus*
ANSP 14647	*Neotamias minimus atristriatus*
ANSP 14638	*Neotamias minimus atristriatus*
ANSP 14643	*Neotamias minimus atristriatus*

**TABLE A3:** Final identification key for differentiating *Neotamias minimus atristriatus* and *Neotamias canipes* using photographs

Pelage trait	*Neotamias minimus atristriatus*	*Neotamias canipes*
Post auricular patches: small patches of lighter fur directly posterior to ears	Small and darker	Larger, prominent and white
Lower face: lighter patch below lowest dark stripe	Dingy or yellowish	Whitish or clean pale gray
Lower light face stripe: light stripe below eye that goes to ear	Grayish or dingy	White
Upper light face stripe: light stripe/patch above eye	Less white, less prominent	White
Shoulder	Yellowish, orange, darker, more intense	Grayer, lighter, less intense
Dark outer stripes: there are five dark dorsal stripes—this refers to the pair of outermost stripes, and these stripes may be indistinct	Blacker; narrower and more distinct (looks like it was drawn on with a marker)	Browner; wider and less distinct (looks like it was painted on with a brush)
White outer stripes: there are four light stripes—this refers to the pair of outermost light stripes	Dingy mixed with brown hairs	White
Dark median stripes: the pair of dark stripes immediately lateral to the middle dark stripe	Darker, thin, blackish (looks like it was drawn on with a marker)	Thick, brownish (looks like it was painted on with a brush)
Dark stripes on rump: this character describes whether the pair of dark median stripes changes color over the rump	The pair of dark median stripes remains dark and distinct all the way down over the rump to near the base of the tail	The pair of dark median stripes changes color posteriorly, becoming a lighter brown and may become so indistinct as to disappear
Hip	Yellower/more orange	Gray
Dorsal hindfoot	Pale yellowish orange	Yellowish gray
Dorsal tail	Hairs mixed black and orange	Hairs mixed black and white
Belly	Light beige, yellowish or orange; darker	Creamy or white or gray; lighter; may have an orange tint
Underside of back leg	More orange	white/gray, may have an orange tint
Underside of front leg	Orange	White/gray

## APPENDIX B

### Field validation survey techniques

#### B.1 Live trapping

We deployed Sherman live traps in meandering lines of 30–40 traps spaced 3–5 m apart. In 2018, we also deployed traps as arrays of 17 traps spaced 5 m apart on 4 perpendicular transects radiating from camera‐trap sites. We baited traps with oats and peanut butter. Live trap surveys lasted from 2 to 4 days for a given trap array or trap line. For all chipmunks captured, we collected data on tail length, hind foot length, ear length, mass, sex, and reproductive status. We identified captured chipmunks based on the external quantitative measurements (Frey, 2010). If a species was captured at least once at a field validation study area, we considered the species to have been detected in that area via live trapping. Small mammals were captured and handled in accordance with New Mexico scientific collecting permit (2868) issued to J.K. Frey. Field methods followed those recommended by the American Society of Mammalogists (Sikes et al., 2016) and as approved by the New Mexico State University Institutional Animal Care and Use Committee (number 2018‐005).

#### B.2 Camera trapping

Camera traps were deployed as part of a range‐wide occupancy study, and 105 of the camera‐trap sites were located within the 9 field validation study areas. At each site, a remote camera (Reconyx PC800 HyperFire) was mounted vertically approximately 45 cm above the ground using a PVC frame (Appendix C). The camera trap was baited with peanut butter placed inside a PVC tube with holes to allow scent to escape and staked to the ground in front of the camera (Perkins‐Taylor & Frey, 2018). The number of survey days varied among camera sites from 3 to 16 days.

Laboratory assistants identified animals in camera‐trap photographs and tagged all photographs of chipmunks to genus for further identification. All chipmunk photographs were identified to species with an associated confidence‐rank by two or three trained observers. We considered multiple consecutive photographs as a series of the same individual when assigning species, and all photographs in a series received the same identification, unless multiple chipmunks were clearly present in the series. If more than one minute passed between consecutive photographs, we considered a photograph to be part of a new series. We managed all photograph metadata using the Colorado Parks and Wildlife Photo Warehouse Microsoft Access application (Newkirk, 2016).

## APPENDIX C

### Camera stand construction and deployment

#### C.1 Camera stand and bait tube construction

The camera mounting stands followed a tripod design, made from three meter‐long pieces of ½” PVC pipe and a ½” PVC three bend elbow joint. The stands held the cameras approximately 45 cm above the ground. The cameras secured to the stands using the threaded insert on the back of the camera housing, and cameras were angled downwards of horizontal, pointed at bait tubes (Figure C1). We designed the camera stands to be lightweight, quick to deploy, and easily hidden from the public.

For each mount, we used 3 meters of ½” PVC pipe, one ½” PVC side outlet 90‐degree elbow joint with a three bend design, two ¼” by 5” eyebolts, three ¼” nuts, a 3/8” hex bolt, a 3/8” nut, and three 3/8” washers.

To build the tripod, the ½” PVC pipe was cut into 1 meter lengths using a PVC pipe cutter. One end of each section of pipe was cut at an angle, allowing us to drive the end of the tripod leg into the dirt if necessary, and a hole was drilled near the end, so that the leg could be pegged into the ground using a tent stake (Figure  C2). The PVC pipes and PVC elbow joints were spray‐painted in green and brown colors, to provide camouflage from the public (Figures C1 and C2).

To affix the cameras to the tripods, we built easily adjustable camera attachments. We used a 7/32 drill bit to drill holes down through the PVC elbow joint, and we screwed an eyebolt through the elbow joint, with a ¼” nut at the top of the elbow joint and a ¼” washer and nut at the bottom of the elbow joint (Figure C3a). Next, we ran the 3/8” hex bolt through the loops of both eyebolts, with a 3/8” washer between the eyebolts and fastened a 3/8” washer and 3/8” nut on the end of the bolt (Figure C3b). A ¼” nut was screwed onto the end of the free eyebolt (Figure C3c).

We made the bait tubes using ½” Charlotte PVC pipe. We cut the PVC pipe into 4” pieces using a PVC pipe cutter and drilled ¼” holes along the tubes (Figure C4).

#### C.2 Camera deployment

When deploying cameras in the field, we fit the tripod legs into the three outlets of the elbow joint. We screwed the end of the free eyebolt into the threaded insert at the back of the camera housing and tightened the ¼” nut down against the back of the camera housing to hold the camera securely in place. The angle of the camera was easily adjusted by loosening the 3/8” hex bolt and nut and by loosening the nut that sat at the top of the elbow joint. When the camera angle was satisfactory, we tightened down all nuts and bolts, to secure the camera in place (Figure C1). We used tent stakes to peg the legs of the tripod to the ground, sometimes driving a tripod leg into the dirt when deploying a camera on sloped terrain.

We used peanut butter to bait the camera traps. We put a spoonful of peanut butter onto a gauze pad and then wrapped the peanut butter up and inserted it into the bait tube (Figure C4). We used a tent stake or a stick to shove the gauze packet into position halfway through the bait tube. Because the gauze packets were tightly packed into the bait tubes, they were inaccessible to animals. We then pegged the bait tube to the ground in the field of view of the camera using a tent stake.

## APPENDIX D

### Specimen identification by experts, for the development of the identification key

**TABLE D1:** Percentage of photographs of nonants of specimens correctly identified as *N. m*. *atristriatus* (*N* = 28) or *N*. *canipes* (*N* = 28) by confidence level during testing of a preliminary identification key by two experts (see Appendix A1 for preliminary identification key)

Confidence rating	Correct *Neotamias minimus atristriatus* identification	Correct *Neotamias canipes* identification	Correct species identification
No confidence (1)	41%	88%	68%
Not very confident (2)	70%	96%	82%
Somewhat confident (3)	95%	99%	97%
Very confident (4)	100%	100%	100%
Somewhat to very confident (3 or 4)	97%	100%	98%

**TABLE D2:** Percentage of photographs of nonants of specimens of *Neotamias minimus atristriatus* and *Neotamias canipes* correctly identified to species, by two experts during testing of a preliminary identification key for distinguishing between the species (see Appendix A1 for preliminary identification key)

Nonant	McKibben correct identifications	Frey correct identifications	Overall correct identifications
Middle dorsal	91%	93%	92%
Posterior lateral	95%	91%	93%
Posterior ventral	88%	68%	78%
Anterior ventral	96%	96%	96%
Middle ventral	93%	89%	91%
Anterior lateral	89%	96%	93%
Posterior dorsal	89%	89%	89%
Anterior dorsal	95%	93%	94%
Middle lateral	89%	91%	90%

**TABLE D3:** Misidentification rates for pelage traits by two experts identifying photographs of nonants of specimens of *Neotamias minimus atristriatus* (*N* = 28) and *Neotamias canipes* (*N* = 28), during testing of a preliminary identification key (see Appendix A1 for preliminary identification key)

Trait	McKibben	Frey	Overall
Post auricular patches	6.56%	2.73%	4.09%
Lower face	5.36%	3.64%	4.22%
Lower light face stripe	6.74%	3.92%	5.24%
Upper light face stripe	6.12%	3.64%	4.81%
Crown	15.38%	6.58%	8.82%
Shoulder	5.71%	2.27%	3.25%
Dark outer stripes	5.02%	3.29%	4.05%
White outer stripes	5.26%	3.89%	4.45%
Dark median stripes	2.16%	5.76%	4.27%
Dark stripes on rump	1.37%	3.68%	2.59%
Hip	9.86%	7.45%	8.19%
Dorsal hindfoot	6.48%	5.81%	6.19%
Dorsal tail	5.61%	9.09%	7.28%
Ventral tail	12.50%	29.23%	21.49%
Belly	5.36%	13.69%	9.52%
Underside of back leg	6.06%	17.71%	12.96%
Underside of front leg	3.45%	2.67%	3.01%

**TABLE D4:** Error matrix showing the true species identification versus the assessment of species identification by two expert observers based on images of single nonants, identified using the preliminary identification key to identify *Neotamias minimus atristriatus* and *Neotamias canipes* (see Appendix A1 for identification key)

		True species identification			
		*N. m. atristriatus*	*N. canipes*	Row total	
Identification by expert observers using preliminary identification key	*N. m. atristriatus*	490	79	504	
	*N. canipes*	14	425	504	
	Column total	569	439	1,008	
	Accuracy by species	490/569 = 86.1%	425/439 = 96.8%		Overall accuracy = 915/1,008 = 90.7%

## APPENDIX E

### Specimen identification by nonexperts.

**TABLE E1:** Error matrices comparing the true species identification versus the assessment of species identification by untrained, partially trained, and fully trained observers identifying *Neotamias minimus atristriatus and Neotamias canipes* from photographs of specimens. Untrained observers used literature references (A and B); partially trained observers used the identification key (C); and fully trained observers used the identification key and completed a training program (D)

A)		True species identification			
		*N. m. atristriatus*	*N. canipes*	Row total	
Identification by untrained observers, unbalanced set	*N. m. atristriatus*	101	90	191	
	*N. canipes*	95	94	189	
	Column total	196	184	380	
	Accuracy by species	101/196 = 51.5%	94/184 = 51.1%		Overall accuracy = 195/380 = 51.3%

B)		*N. m. atristriatus*	*N. canipes*	Row total	
Identification by literature observers	*N. m. atristriatus*	154	47	201	
	*N. canipes*	36	143	179	
	Column total	190	190	380
	Accuracy by species	154/190 = 81.0%	143/190 = 75.2%		Overall accuracy = 297/380 = 78.2%

C)		*N. m. atristriatus*	*N. canipes*		
Identification by key observers, before training	*N. m. atristriatus*	140	11	141	
	*N. canipes*	10	139	139	
	Column total	150	150	280	
	Accuracy by species	140/150 = 93.3%	139/150 = 92.6%		Overall accuracy = 279/300 = 93.0%

D)		*N. m. atristriatus*	*N. canipes*		
Identification by key observers, after training	*N. m. atristriatus*	417	8	425	
	*N. canipes*	3	412	415	
	Column total	420	420	840	
	Accuracy by species	417/420 = 99.3%	412/440 = 98.1%		Overall accuracy = 829/840 = 98.7%

**TABLE E2:** Overall accuracy and accuracy by species for fifteen trainees using the identification key (see Appendix Table A3 for identification key), before and after completing a training program for identifying specimens of *Neotamias minimus atristriatus* and *Neotamias canipes* based on photographs

	Before training			After training		
Observer ID	Overall accuracy	*N. m. atristriatus*	*N. canipes*	Overall accuracy	*N. m. atristriatus*	*N. canipes*
1	95%	90%	100%	96%	96%	96%
2	100%	100%	100%	100%	100%	100%
3	95%	100%	90%	100%	100%	100%
4	95%	100%	90%	98%	100%	96%
5	85%	80%	90%	98%	100%	96%
6	100%	100%	100%	100%	100%	100%
7	85%	80%	90%	96%	96%	96%
8	85%	90%	80%	100%	100%	100%
9	90%	90%	90%	100%	100%	100%
10	100%	100%	100%	100%	100%	100%
11	90%	90%	90%	95%	96%	93%
12	100%	100%	100%	100%	100%	100%
13	85%	80%	90%	96%	100%	93%
14	100%	100%	100%	100%	100%	100%
15	90%	100%	80%	100%	100%	100%
Total	93%	93%	93%	99%	99%	98%

**TABLE E3:** Misidentification rates for pelage traits by species and overall for fifteen trainees during training set 1 (504 photographs of nonants of specimens of *Neotamias minimus atristriatus* and *Neotamias canipes*), while training on the use of the final identification key (see Appendix Table A3 for identification key)

Trait	*N. canipes*	*N. m. atristriatus*	Overall
Post auricular patches	19.01%	19.64%	19.32%
Lower face	20.76%	22.07%	21.44%
Lower light face stripe	20.11%	22.58%	21.34%
Upper light face stripe	17.83%	20.84%	19.31%
Shoulder	16.47%	19.65%	18.13%
Dark outer stripes	16.19%	18.01%	17.11%
White outer stripes	17.03%	19.92%	18.48%
Dark median stripes	20.34%	17.40%	18.83%
Dark stripes on rump	19.67%	15.86%	17.72%
Hip	14.48%	23.27%	18.96%
Dorsal hindfoot	15.28%	14.77%	14.99%
Dorsal tail	20.03%	15.47%	17.78%
Belly	13.28%	16.54%	14.90%
Underside of back leg	15.53%	12.68%	14.01%
Underside of front leg	11.69%	19.42%	15.55%

## APPENDIX F

### Identification of a series of photographs of specimens when one species is rare.

#### E.1 Introduction

In situations where similar looking species co‐occur, often one species is common in the dataset, while the other is rare. False‐positive and false‐negative rates are higher with rare species, likely because people are eager to report rare species or because people are skeptical of identifications of rare species (Farmer et al., 2012; Mckelvey et al., 2008; Swanson et al., 2016). Additionally, an observer identifying a small series of animals will have less opportunity to learn the rarer species because it will be encountered less frequently.

Because literature observers in our study were asked to identify a set of photographs that was divided evenly between the two species, observers were likely able to learn during the identification process. We predicted that observers identifying an unbalanced set of slides would have lower accuracy than observers identifying a balanced set of slides. To test this, we compared the accuracy of literature observers on unbalanced sets of slides to the accuracy of literature observers during our main study, who were tested on balanced sets of slides.

#### E.2 Methods

We tested whether observer accuracy was affected by the rarity of a species within the dataset by comparing the accuracy of literature observers identifying a balanced set of slides (hereafter “balanced‐literature observers”) to that of literature observers identifying an unbalanced set of slides (hereafter “unbalanced‐literature observers”). The methods for testing the balanced‐literature observers are reported in the main text (section 2.2). We provided the unbalanced‐literature observers (*N* = 19) with the same identification resources as the balanced‐literature observers and with a test that consisted of an unbalanced set of slides. We created unbalanced tests by randomly drawing from a sample of 56 slides as well as intentionally skewing to more extreme imbalance; the ratios ranged from 1:19 to 10:10, and we tested 11 observers on random draws and 8 observers on intentionally skewed mixes. We did not tell observers which test they received, because researchers identifying species from camera‐trap photographs do not know the ratio of species in their dataset.

We used Welch's unequal variances one‐tailed *t* test at a .05 significance level to test whether the accuracy of literature observers identifying an unbalanced set was lower than the accuracy of literature observers identifying a balanced set. For the unbalanced‐literature observers, we calculated identification accuracy by confidence‐rank and Pearson's correlation coefficient (*r*) to test for a correlation between confidence‐rank and accuracy.

#### E.3 Results

Literature observers identifying an unbalanced set of slides had high misidentification rates, roughly equivalent to flipping a coin (51.3% accuracy; Table F1). As predicted, there were significantly (*t* = −4.2, *df* = 27.2, *p* < .001) more misidentifications for unbalanced sets (51.3%) versus balanced sets (78.2% accuracy; Table 1 in the manuscript). For unbalanced‐literature observers, identification accuracy and confidence‐rank were negatively correlated (*r* = −0.30) and identifications made with very high confidence had accuracy worse than random (<50%).

**TABLE F1:** Error matrix showing the true species identification versus the assessment of species identification by nineteen untrained observers, using materials in the literature to identify *Neotamias minimus atristriatus* and *Neotamias canipes* (see main text). Each observer was given a randomized and unbalanced series of the two species

		True species identification			
		*N. m. atristriatus*	*N. canipes*	Row total	
Identification by literature observers, identifying unbalanced sets	*N. m. atristriatus*	101	90	191	
	*N. canipes*	95	94	189	
	Column total	196	184	380	
	Accuracy by species	101/196 = 51.5%	94/184 = 51.1%		Overall accuracy = 195/380 = 51.3%

**TABLE F2:** Accuracy of identification of *Neotamias minimus atristriatus* and *Neotamias canipes* from photographs of verified museum specimens at different observer reported confidence‐ranks for literature observers identifying an unbalanced set of slides

Observer confidence	Number of identifications	Accuracy
No confidence (1)	35	51.4%
Not very confident (2)	91	47.3%
Somewhat confident (3)	150	54.7%
Very confident (4)	83	45.8%

## APPENDIX G

### Estimate of hours required for key development, testing, and training

**TABLE G1:** Estimate of the hours required to develop an identification key, train observers, and test the efficacy of the key for differentiating between *Neotamias minimus atristriatus* and *Neotamias canipes* in camera‐trap photographs (see Appendix Table A3 for identification key)

Action	Person	Number of people	Hours per person	Total hours
Examine verified specimens	Primary investigator	2	3	6
Create key based on external characteristics	Primary investigator	2	6	12
Photograph museum specimens and create key tests	Technician	1	100	100
Test key to ensure it is possible to differentiate species with a reasonable level of accuracy	Primary investigator	2	12	24
Revise key based on test results	Primary investigator	2	8	16
Train observers on use of key	Technician	3	12	36
Test observers on identifications with confidence rankings	Technician	3	1	3
Grand total				198
